# Palladium nanoparticle-decorated multi-layer Ti_3_C_2_T_*x*_ dual-functioning as a highly sensitive hydrogen gas sensor and hydrogen storage †

**DOI:** 10.1039/d0ra10879k

**Published:** 2021-02-15

**Authors:** Thanh Hoang Phuong Doan, Won G. Hong, Jin-Seo Noh

**Affiliations:** Department of Physics, Gachon University 1342 Seongnamdaero, Sujeong-gu Seongnam-si Gyeonggi-do 13120 Korea jinseonoh@gachon.ac.kr +82 317505611; Research Center for Materials Analysis, Korea Basic Science Institute (KBSI) Daejeon 34133 Korea

## Abstract

In this work, palladium nanoparticle (PdNP)-decorated Ti_3_C_2_T_*x*_ MXene (Pd–Ti_3_C_2_T_*x*_) was synthesized by a simple two-step process. For this, multilayer Ti_3_C_2_T_*x*_ MXene (ML-Ti_3_C_2_T_*x*_) was first prepared by a selective HF etching technique, and PdNPs were directly grown on the surface of ML-Ti_3_C_2_T_*x*_ flakes using a polyol method. The relative weight fraction of PdNPs to ML-Ti_3_C_2_T_*x*_ was elaborately controlled to derive the optimal size and distribution of PdNPs, thereby to maximize its performance as a hydrogen sensor. The optimized Pd–Ti_3_C_2_T_*x*_ nanocomposite showed superb hydrogen-sensing capability even at room temperature with sharp, large, reproducible, concentration-dependent, and hydrogen-selective responses. Furthermore, the nanocomposite also unveiled some extent of hydrogen storage capability at room temperature and 77 K, raising a possibility that it can dual-function as a hydrogen sensor and hydrogen storage.

## Abstract 2

Nanocomposites of PdNPs and ML-Ti_3_C_2_T_*x*_ MXene are synthesized using a facile two-step process, and it is demonstrated that they can dual-function as a highly sensitive hydrogen sensor and hydrogen storage.

## Graphical abstract

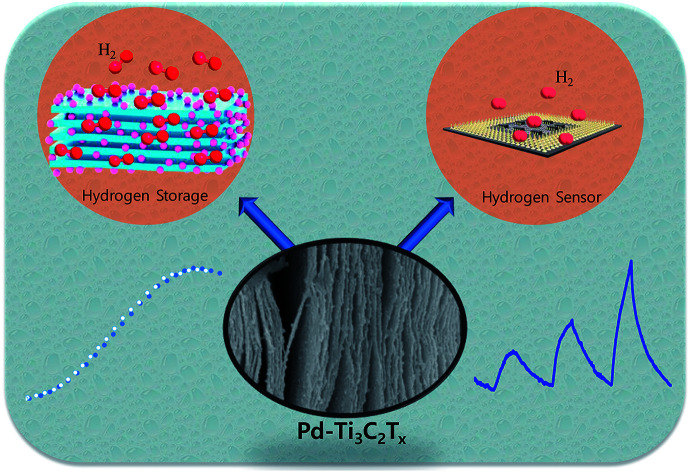

## 1. Introduction

Nowadays, human activities produce an ever-increasing amount of greenhouse gases, which are generated mostly from the use of conventional fossil fuels. In order to reduce the emission of greenhouse gases, many countries around the world have strived to find alternative fuels, which are renewable and environment-friendly. Hydrogen gas (H_2_) is a promising fuel for energy generation due to its cleanliness, abundance, and recyclability.^1^ For this reason, H_2_ has recently emerged as a hot research topic, and is commercialized in various sectors such as transportation and local power generation.^1,2^

The major challenges to tackle for the dissemination of hydrogen fuel include discovering a means to store hydrogen with high capacity and securing the safety from its potential leak.^3–5^ In particular, the safety issue is important because H_2_ is extremely flammable if its concentration is higher than 4% in air.^6^ Moreover, H_2_ is colorless and odorless, but very diffusive. These attributes underscore the importance of quick and sensitive detection of hydrogen leaks. Many hydrogen sensors have been developed towards good sensitivity and short response time, employing various nanomaterials.^4^ However, several drawbacks such as high operating temperature and complicated fabrication procedures still need to be improved.^7–11^ In this regard, a continued search for the better sensing materials and processing routes are necessary. Palladium (Pd) is one of the most popular H_2_-sensing materials due to its unique reaction with H_2_. Despite the good H_2_ selectivity and sensitivity, the response of bulk Pd is limited and it shows some brittleness when exposed to H_2_ repeatedly. To improve its response and relieve H_2_ brittleness, Pd nanostructures have been synthesized and further hybridized with other nanomaterials, including ZnO nanorods (NRs), SnO_2_ nanowires (NWs), graphene oxide (GO), and reduced graphene oxide (rGO).^12–14^ Among such nanomaterials, 2D materials attract renewed attention as a sensing platform due to their large surface area and directional charge transport. Other than carbon-based materials, MXenes are a class of noble 2D materials with intriguing structure and properties. Multi-layer Ti_3_C_2_T_*x*_ MXene (ML-Ti_3_C_2_T_*x*_) has been widely used for gas sensing, owing to its facile synthesis route and strong interaction with gaseous molecules.^15^ In addition, it is highly conductive electrically, and its unique structure favors to reduce the electron transportation path distance.^16^ Therefore, combining the outstanding virtues of nanostructured Pd and ML-Ti_3_C_2_T_*x*_ may be an elaborate strategy to achieve high-performance H_2_ gas sensors.

Another challenge for the expanded use of H_2_ energy is to develop a safe hydrogen storage with high capacity. Today, compressing H_2_ under high pressure is the most conventional technology due to its cost advantage. However, the H_2_ storage capacity of the technology falls behind the general need, and a large volume or weight is required to contain enough H_2_.^17^ To address this issue, solid state storage media have been developed, including elemental metals, various alloys with formulas of AB, AB_2_, and AB_5_, alanates, and carbon materials.^18^ Pd can store H_2_*via* PdH formation and supply H atoms to nearby medium by so-called “spill-over” mechanism.^19^ Moreover, the reactive termination groups and unique layered structure of ML-Ti_3_C_2_T_*x*_ may let this material considered for hydrogen storage.^20^

In this work, we developed a highly sensitive and selective hydrogen gas sensor by decorating Pd nanoparticles (PdNPs) on the surface of ML-Ti_3_C_2_T_*x*_. Furthermore, it was demonstrated that the hydrogen gas sensor could also function as hydrogen storage. The dual functioning of the PdNPs-decorated ML-Ti_3_C_2_T_*x*_ (Pd–Ti_3_C_2_T_*x*_) would give new insights into active hydrogen gas sensors, which can accommodate part of leaked H_2_ gas, thereby alleviating the potential explosion.

## 2. Experimental section

### 2.1 Materials

Ethylene glycol (C_2_H_6_O_2_, EG), polyvinylpyrrolidone (PVP, *M*_w_ ∼1 300 000), sodium tetrachloropalladate (ii) (Na_2_PdCl_4_) were all purchased from Sigma-Aldrich (St. Louis, MO, USA). Ti_3_AlC_2_ powder (400 mesh) was purchased from 11 Technology Co., Ltd (Changchun, China). Hydrofluoric acid (HF, ∼50%) was purchased from Fisher Scientific (Fair Lawn, NJ, USA). Ethyl alcohol (C_2_H_5_OH) were purchased from Daejung Chem (Siheung, South Korea).

### 2.2 Synthesis of ML-Ti_3_C_2_T_*x*_ and Pd–Ti_3_C_2_T_*x*_

**Fig. 1:**
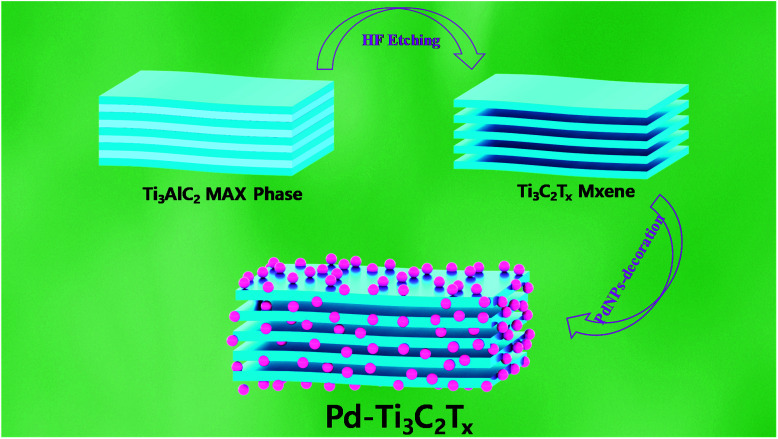
Schematic illustration of the sequential process for fabrication of ML-Ti_3_C_2_T_*x*_ and Pd–Ti_3_C_2_T_*x*_.

Fig. 1 depicts the whole process for the fabrication of Pd–Ti_3_C_2_T_*x*_. First of all, ML-Ti_3_C_2_T_*x*_ was synthesized using the same methods with minor modifications as already published literatures.^21,22^ Al layers of Ti_3_AlC_2_ MAX phase were selectively etched using 50% HF. In more detail, a 4 g of Ti_3_AlC_2_ powder was slowly added to a plastic bottle containing 100 ml of HF, which was placed in an ice bath. Then, the colloidal solution was kept for 24 h at 50 °C under continuous stirring. At the next step, the etched product was washed using ethanol until the pH reached around 6. Finally, it was dried for 6 h in a convection oven at 60 °C.

In order to decorate PdNPs on the surface of ML-Ti_3_C_2_T_*x*_, we modified the process reported in the previous literatures.^23,24^ Firstly, ML-Ti_3_C_2_T_*x*_ flakes were dispersed over 10 min in a 50 ml vial containing 10 ml EG under ultrasonication, then this vial was placed in an oil bath at 160 °C for 2 h with stirring. Here, the weight of ML-Ti_3_C_2_T_*x*_ was controlled from 30, 60, to 90 mg. (from M1 to M3 sample, respectively). In the meantime, 8 ml of PVP solution (9.5 mM) and 4 ml of Na_2_PdCl_4_ solution (3.5 mM) were independently prepared using EG as a solvent. Next, these solutions were slowly injected into the ML-Ti_3_C_2_T_*x*_ colloidal solutions over a span of 30 min, followed by continuous stirring for additional 5 min. At the last step, the reaction products were washed 4 times using ethanol and dried for 6 h at 60 °C. The Pd–Ti_3_C_2_T_*x*_ samples were named M1 (30 mg), M2 (60 mg), and M3 (90 mg), respectively, depending on the weight of ML-Ti_3_C_2_T_*x*_ used for the nanocomposite formation.

### 2.3 Fabrication of hydrogen gas sensors

A silicon (Si) substrate of size in 1 cm × 2 cm was rinsed several times with ethanol and isopropyl alcohol (IPA) to remove organic and inorganic dirt on the surface, and then completely dried at 60 °C. At the same time, a 30 mg of Pd–Ti_3_C_2_T_*x*_ powder was dispersed into 5 ml of ethanol under sonication. This colloidal solution was drop-cast onto the surface of pre-cleaned Si substrate to form a H_2_-sensing film, then dried in an oven at 60 °C. Two contacts were made on the film using silver (Ag) paste for subsequent gold (Au) wiring to external electric units.

### 2.4 Material characterization and gas-sensing tests

The morphologies of raw materials and Pd–Ti_3_C_2_T_*x*_ samples were investigated using a field emission scanning electron microscope (FE-SEM, JEOL JSM-7500F) mounted with an energy-dispersive X-ray spectrometer (EDX). The crystalline characteristics of samples were examined by X-ray diffraction (XRD, X'pert Pro MPD) with copper (Cu) Kα radiation. Furthermore, X-ray photoelectron spectroscopy (XPS, K-Alpha, Thermo Electron) was used to examine the binding states of ML-Ti_3_C_2_T_*x*_ and Pd–Ti_3_C_2_T_*x*_ samples.

A unique-designed gas-sensing system was employed to evaluate the gas-sensing performance of the samples. For it, a sample was loaded into a gas chamber with a capacity of 682 cc and Au-wired to lead pins that were connected to an electrical source and measure unit outside the chamber. The chamber has branched channels with multiple gas sources, including H_2_ gas and synthetic air. The concentrations of target gases were controlled by a gas-mixing system, and the controlled gases were fed into the chamber at 500 cm^3^ min^−1^ using a mass flow controller (MFC). A Keithley 2450 multimeter was used to measure the variation of electrical resistance in response to target gases, and it was recorded in a computer through a LabView program. In this study, all gas-sensing tests were conducted at room temperature using air as a carrier gas. The response of a gas sensor was defined as1
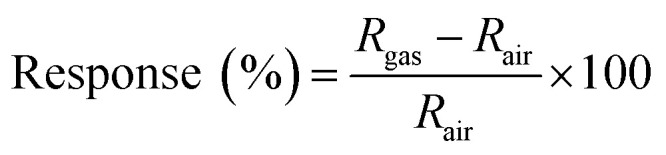
where *R*_air_ and *R*_gas_ are the electrical resistances of the sensor in air and a target gas, respectively.

### 2.5 Hydrogen storage test

The hydrogen storage capability of a Pd–Ti_3_C_2_T_*x*_ nanocomposite was tested using a H_2_ adsorption–desorption measurement system (Belsorp-HP, BEL Japan Inc.). In the test, the hydrogen storage capacity was measured volumetrically with a computer-controlled pressure-composition isotherm. The pressure was gradually increased up to 85 bar, and 99.9999% of H_2_ gas was used in all the measurements. The test was conducted at both room temperature and 77 K. To increase the credibility of the measurement, the system was calibrated with LaNi_5_ at room temperature, and with activated carbon (surface area ∼3000 m^2^ g^−1^) at 77 K.

## 3. Results and discussion

### 3.1 Morphologies and compositions

**Fig. 2:**
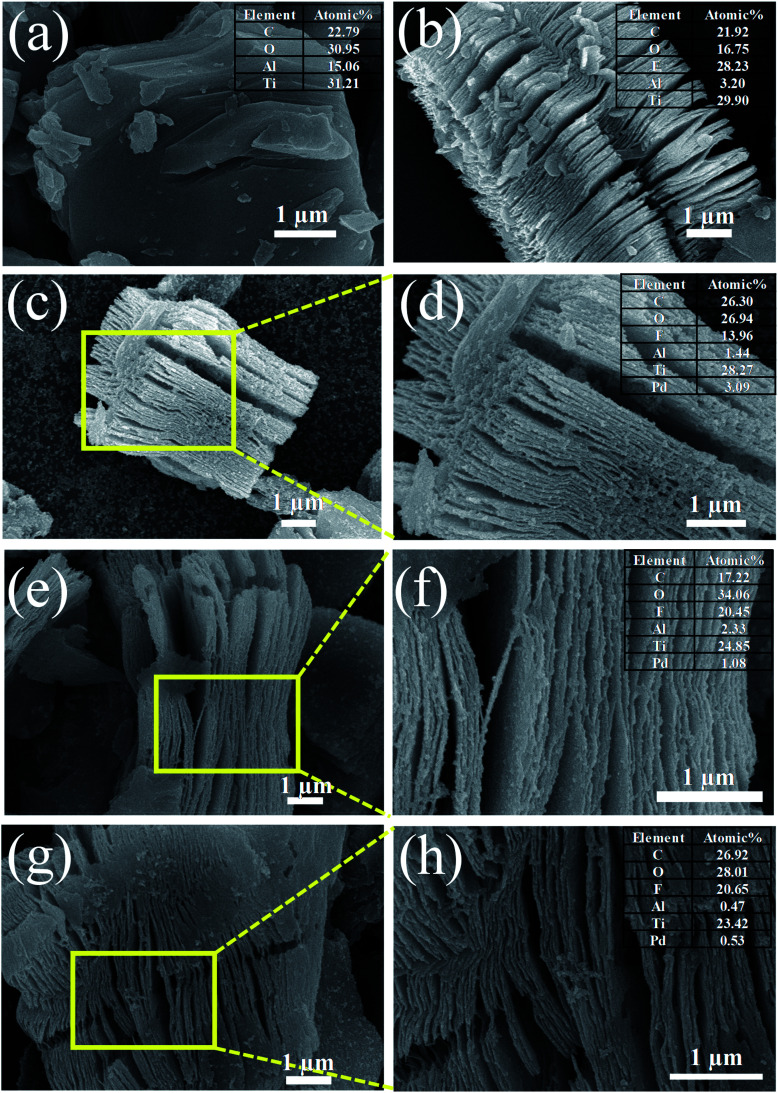
SEM images of (a) Ti_3_AlC_2_ MAX phase, (b) ML-Ti_3_C_2_T_*x*_, (c and d) M1, (e and f) M2, and (g and h) M3 samples. The insets show the EDX elemental compositions of the respective samples.

Fig. 2 shows the SEM images of Ti_3_AlC_2_ MAX phase, ML-Ti_3_C_2_T_*x*_ MXene, and Pd–Ti_3_C_2_T_*x*_ nanocomposites. As can be seen in Fig. 2(a), Ti_3_AlC_2_ MAX phase is composed of microsheets, the side surfaces of which reveal slightly laminated pattern. After HF etching, the morphology is fully developed to an accordion-like laminated structure and Al content is greatly reduced to 3.2 at%, demonstrating the successful transformation from the MAX phase to ML-MXene (Fig. 2(b)). The atomic ratio of Ti to C is estimated at 1.36 from the SEM-EDX analysis (see the inset of Fig. 2(b)), which is close to the stoichiometric composition of Ti_3_C_2_T_*x*_ MXene. It is also found that a large amount of F termination group (28.23 at%) was formed during the HF etching process. Fig. 2(c)–(h) present the SEM images of Pd–Ti_3_C_2_T_*x*_ nanocomposites (M1, M2, and M3 samples in sequence). It is clear that PdNPs are evenly distributed on the side surfaces and clevaged surfaces of ML-Ti_3_C_2_T_*x*_ for all the samples. However, more detailed distribution turned to be dependent on the relative content of Pd precursor. For M1 sample (Pd content = 3.09 at%), free PdNPs that are not stuck to the Ti_3_C_2_T_*x*_ surface appear, and many PdNPs observed in between neighboring layers seem to be agglomerated. These may originate from the overdosed Pd precursor, and weaken the advantage of the layer-structured MXene, leading to the deterioration of its gas-sensing performance. On the contrary, both the size and density of PdNPs seem to be insufficient for M3 sample (Pd content = 0.53 at%), which most likely result from the underdosed Pd precursor. M2 sample can be singled out as the best sample from every aspect like the size, density, and distribution of PdNPs. For this sample, the Pd content is estimated at 1.1 at%. The uniform distribution of major elements in M2 sample can be found from SEM-EDX element maps (see Fig. S1†).

### 3.2 Crystal quality and binding states

**Fig. 3:**
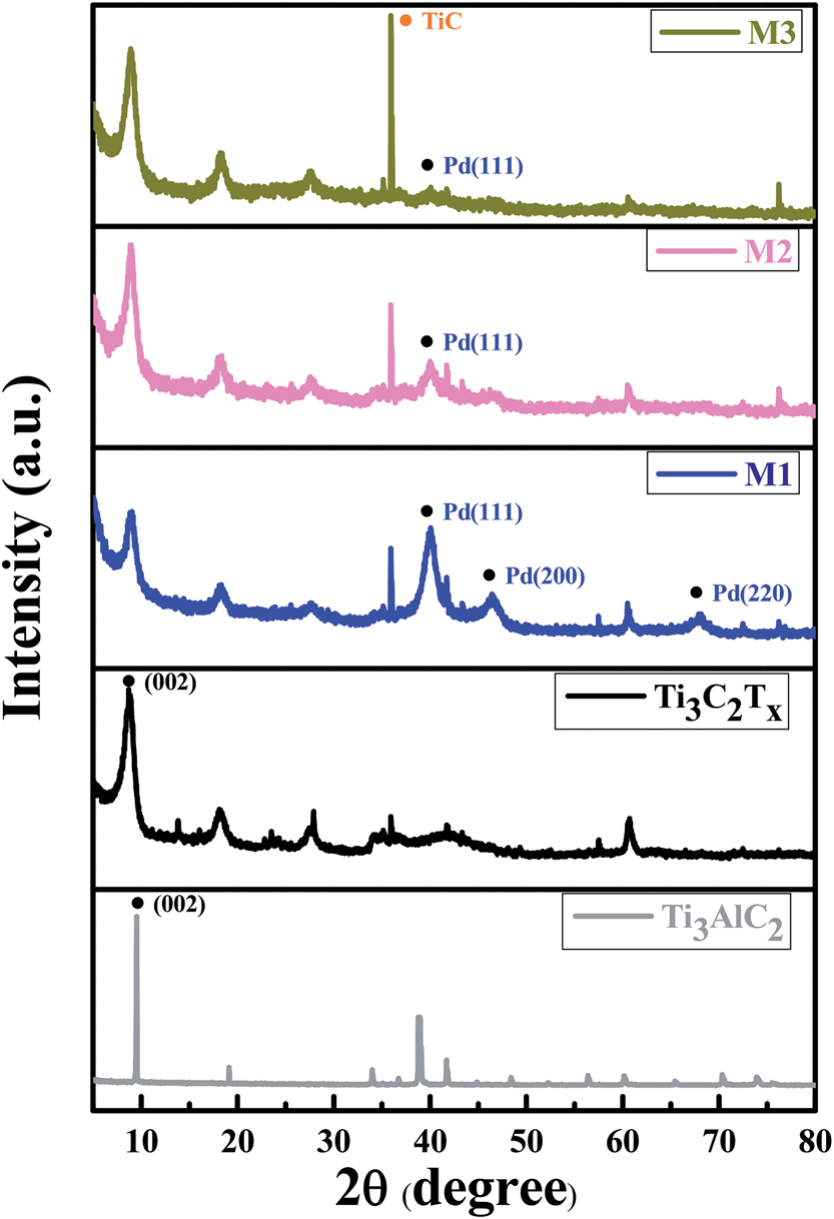
XRD patterns of Ti_3_AlC_2_ MAX phase, ML-Ti_3_C_2_T_*x*_, and three nanocomposite samples (M1, M2, and M3).

In order to examine the crystal quality of the samples, XRD analysis was performed. Fig. 3 exhibits the XRD patterns of Ti_3_AlC_2_ MAX phase, ML-Ti_3_C_2_T_*x*_ MXene, Pd–Ti_3_C_2_T_*x*_ nanocomposites (M1, M2, and M3) from bottom to top. For ML-Ti_3_C_2_T_*x*_, the strong peakest peak is observed at 2*θ* = 8.7°, which is indexed to (002) plane of 2D MXene. This is shifted by 0.82° from the (002) peak position of its parent material, Ti_3_AlC_2_ MAX phase. Moreover, the peak widths were clearly broadened after transforming the MAX phase to ML-MXene. These XRD peak shift and broadening are typical signals representing the full transformation of MAX phase to 2D MXene.^25,26^ Once PdNPs are decorated on the surface of ML-Ti_3_C_2_T_*x*_, Pd peaks appear along with the MXene peaks. For instance, a peak found at 2*θ* = 40.1° is assigned to (111) plane of fcc Pd (JCPDS card no. 05-0681).^27^ However, the peak intensity of M1 sample looks excessive as compared to the (002) intensity of MXene, representing PdNPs are overly decorated, which is the same conclusion as derived from SEM observations. In contrast, the (111) peak intensity is too weak for M3 sample, while a TiC peak appears at 2*θ* = 35.94° as the main phase.^28^ The TiC peak intensity tends to increase as the relative weight fraction of ML-MXene increases. Just like the previous conclusion, the M2 sample shows the most desirable XRD pattern.

**Fig. 4:**
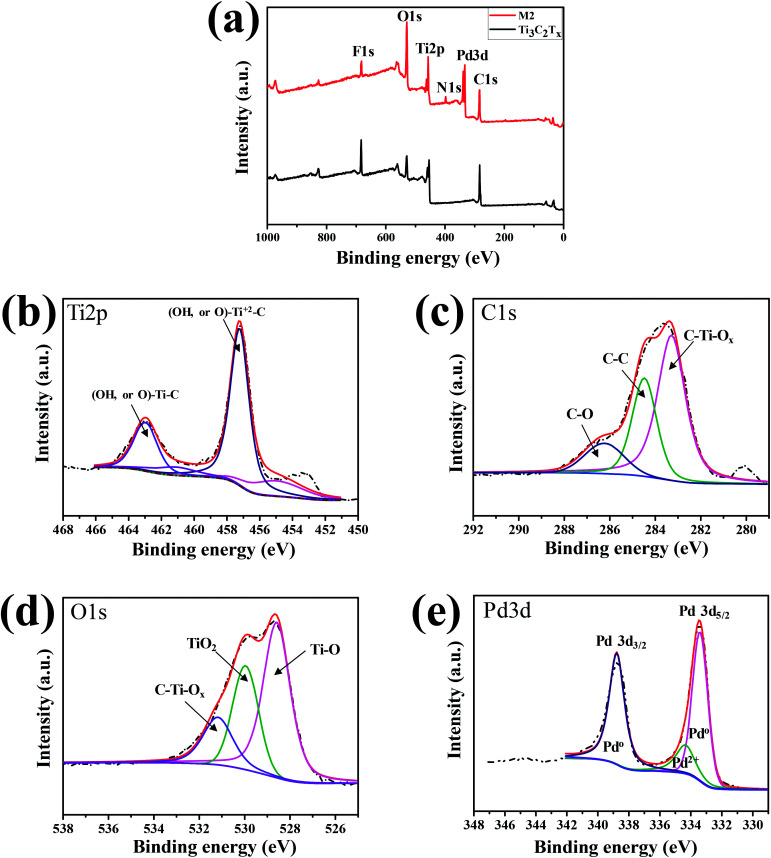
(a) Full XPS spectra of ML-Ti_3_C_2_T_*x*_ and M2 sample. XPS spectra of M2 focused on (b) Ti2p, (c) C1s, (d) O1s, and (e) Pd3d.

The binding states of pure ML-MXene and a nanocomposite sample were analyzed and compared using XPS. Fig. 4(a) shows the XPS full spectra of ML-Ti_3_C_2_T_*x*_ and Pd–Ti_3_C_2_T_*x*_ (M2 sample). Both samples contain C, Ti, O, and F elements, as expected from SEM-EDX data. The clear difference between the two samples can be found from the additional Pd3d and N1s peaks. The Pd3d and N1s peaks are observed only in M2 sample, which are arisen from PdNPs and remanent PVP stabilizer used in the PdNP formation step. The small amount of remanent PVP may accelerate the adsorption and desorption processes of H_2_ molecules due to the reduction of the apparent activation barriers, leading to the improved performance of our hydrogen gas sensors.^29–31^ Element-specific XPS spectra of M2 sample were further analyzed. The Ti2p spectrum in Fig. 4(b) shows two major peaks centered at 463.0 and 457.3 eV, which are assigned to (OH, or O)–Ti–C bond and (OH, or O)–Ti^2+^–C bond, respectively.^32^ This indicate that the ML-Ti_3_C_2_T_*x*_ surface is functionalized by –OH or =O groups after PdNP decoration step. These functional groups may help the nanocomposite to adsorb gas molecules easily. Likewise, the C1s spectrum consists of three main peaks at 283.2, 284.5, and 286.3 eV (Fig. 4(c)), which represent C–Ti–O_*x*_, C–C, and C–O bonds, respectively.^33,34^ This result further supports the presence of surface functional groups. From the fact that the C–O bond is not observed in pure ML-Ti_3_C_2_T_*x*_ MXene (see Fig. S2†), its appearance in M2 sample is inferred to result from the surface oxidation during PdNP decoration. Such bonds as C–O and (OH, or O)–Ti^2+^–C may be responsible for the conductivity decrease observed after decorating PdNPs on ML-Ti_3_C_2_T_*x*_ MXene. Regarding O1s, three peaks are found at 528.6, 530, and 531.2 eV, which correspond to Ti–O, TiO_2_, and C–Ti–O_*x*_, respectively.^32,35^ Furthermore, two sharp Pd3d peaks (Pd3d_5/2_ and Pd3d_3/2_) are observed at the binding energies of 333.5 and 338.8 eV. The energy difference of 5.3 eV between the two peaks is quite close to the previous reports.^25^ Meanwhile, the Pd3d_5/2_ and Pd3d_3/2_ peak positions of the nanocomposite are shifted from those of pure Pd metal (334.88 eV for Pd3d_5/2_ and 340.25 eV for Pd3d_3/2_),^36,37^ due to the interaction of PdNPs and ML-Ti_3_C_2_T_*x*_ MXene.

### 3.3 H_2_-sensing performance and gas selectivity

**Fig. 5:**
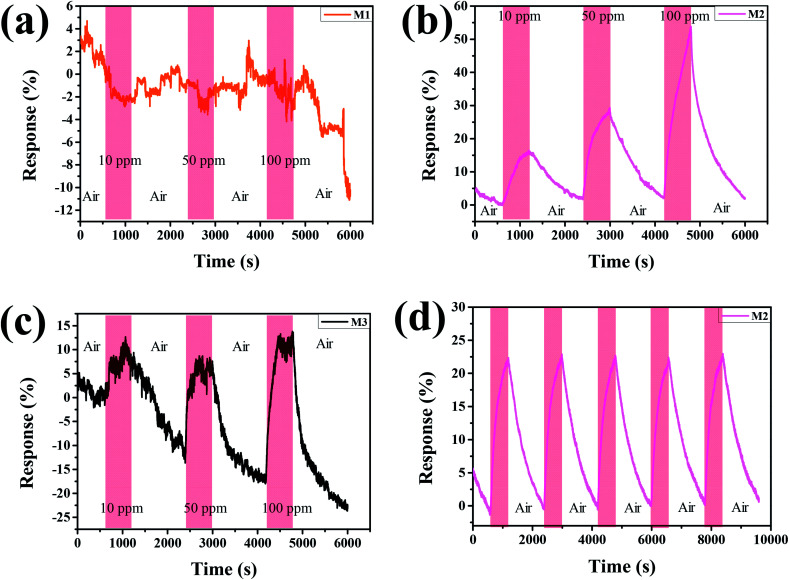
H_2_ concentration-dependent response curves of (a) M1, (b) M2, and (c) M3. (d) Cyclic responses of M2 to a 50 ppm of H_2_ gas.

The H_2_-sensing capability of Pd–Ti_3_C_2_T_*x*_ nanocomposites was evaluated at room temperature. To see the effect of the relative content of PdNPs, we first examined the H_2_-sensing performance of M1, M2, and M3 samples. As displayed in Fig. 5(a), M1 sample exhibits noisy and negative response. The negative response may be attributed to the high density of PdNPs in the sample. PdNPs generally experience a volume expansion on adsorbing H_2_, and can be locally connected when they are spaced close enough, leading to the formation of current path. The high density of PdNPs also have a MXene-screening effect, which limits the involvement of the MXene layer in H_2_-sensing process. On the other hand, both M2 and M3 samples show positive response signals (Fig. 5(b) and (c)), which are related to comparatively lower PdNP densities. Comparing the H_2_-sensing performance of the two samples, M2 is superior to M3 sample in terms of the clarity of signal, magnitude of response, and the degree of recovery. The M2 sample shows clean, large, fully recovered, and completely concentration-dependent response signals. For example, the response of the sample to 100 ppm of H_2_ is calculated to be 56%. In comparison, the rather noisy signal of M3 sample underlines the importance of combining PdNPs and ML-Pd–Ti_3_C_2_T_*x*_ with a golden ratio. Cyclic response test was further performed on the M2 sample, and the result is presented in Fig. 5(d). For this test, a 50 ppm of H_2_ gas was flowed for 10 min followed by 20 min-long air purging, and this cycle was repeated five times. Clear, sharp, and uniformly cyclic response curves are surely observed, demonstrating its excellent H_2_-sensing stability. Furthermore, the H_2_-sensing performance of the best sample (M2) was compared with previous reports in Table 1. It is obvious from the table that our H_2_ sensor has comparative advantages. Of course, some sensors have demonstrated larger responses, but their operating temperatures were in general higher than 100 °C. Moreover, the material combination of Pd and Ti_3_C_2_T_*x*_ MXene has been developed by Zhu *et al.*,^25^ employing a sonication technique of Pd nanocluster and Ti_3_C_2_T_*x*_ MXene suspension. However, its H_2_-sensing response (23%) was smaller than ours, even though a higher concentration of H_2_ (4%) was used for the test. Moreover, our H_2_ sensor shows good long-term stability, as demonstrated in Fig. S3.† Clean and sharp response signals are reproduced even after keeping the sensor for 90 days at ambient condition.

**Table 1:** Comparison of H_2_-sensing performance of some novel gas sensors

Materials	H_2_ concentration	Operating temperature (°C)	Response^a^ (%)	Reference
Pd–Ti_3_C_2_T_*x*_	100 ppm	RT	56	This work
SnO_2_/Pd	100 ppm	300	56	38
MoS_2_–Pt NPs	100 ppm	150	90	39
PdO-decorated p-type CoV_2_O_6_ NPs	30 000 ppm	300	114	40
3D layer-by-layer Pd–Pt–Au	2%	RT	6.94	41
Ti_3_C_2_T_*x*_ MXene@Pd colloidal nanoclusters	4%	RT	23	25
V_2_CT_*x*_ MXene	100 ppm	RT	24	42
Pd/boron nitride/ZnO NWs	10 ppm	200	86	43
Pd/MWCNT	4%	RT	12.3	44

a The responses were read from the figures of publications; they might be not precise.

**Fig. 6:**
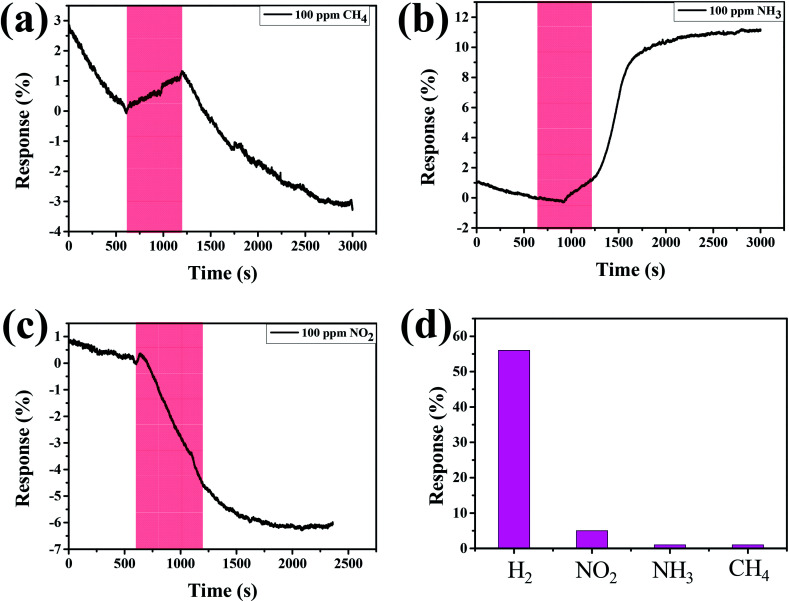
Response curves of M2 sample to 100 ppm of (a) CH_4_, (b) NH_3_, and (c) NO_2_. (d) Comparison of responses of M2 sample to different gases at the concentration of 100 ppm.

In addition, we examined the response behaviors of Pd–Ti_3_C_2_T_*x*_ to other kinds of toxic gases. Fig. 6(a)–(c) show the response curves of M2 sample to 100 ppm of CH_4_, NH_3_, and NO_2_, respectively. For every gas, the response curves are not well developed with small response values, although the sign of response is dependent on the type of gas. The response (∼5%) to NO_2_ gas is slightly larger than the other gases, but the signal is not recovered to its original level after stopping the gas flow. A swift change of response curve is found for CH_4_ gas. However, the response (∼1%) to CH_4_ is too small. Fig. 6(d) compares the responses of M2 to H_2_, NO_2_, NH_3_, and CH_4_ at the fixed concentration of 100 ppm. This comparison manifests that the optimal-designed Pd–Ti_3_C_2_T_*x*_ nanocomposite is well suited for detecting H_2_ gas with high gas selectivity.

**Fig. 7:**
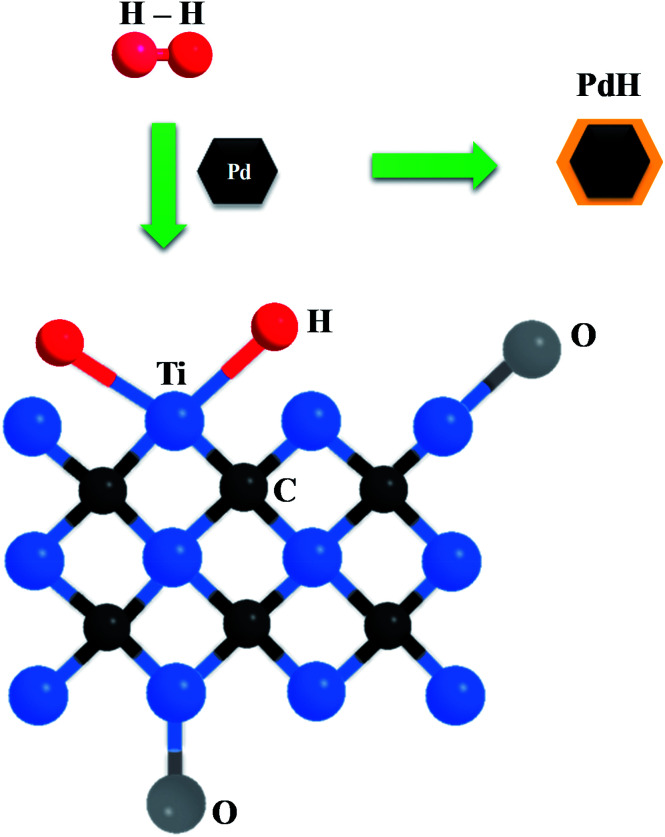
Schematic picture for illustrating the H_2_-sensing mechanism of the Pd–Ti_3_C_2_T_*x*_ nanocomposite.

To explain the superb H_2_-sensing capability of Pd–Ti_3_C_2_T_*x*_, a potential mechanism is suggested. The nanocomposite detects H_2_ gas by the collaborative activities of PdNPs and ML-Ti_3_C_2_T_*x*_, as schematically depicted in Fig. 7. When exposed to H_2_ gas, PdNPs adsorb H_2_ molecules and dissociate them into H atoms, leading to the formation of PdH.^45–47^ This process is facilitated by the catalytic nature of Pd, and consequently increases the material's resistance. PdNPs can also play a role to supply H atoms to nearby ML-Ti_3_C_2_T_*x*_*via* a spill-over mechanism. The transferred H atoms can react with transition metals (Ti in this case) on the surface of ML-Ti_3_C_2_T_*x*_, forming TiH_2_. This leads to a further increase in the sensor resistance. A similar phenomenon has been previously reported in Ti-decorated carbon nanotubes, where dissociated H atoms were adsorbed by Ti atoms without any energy barrier.^48^ When the Pd content is excessive (M1 sample), the role of ML-Ti_3_C_2_T_*x*_ is limited, whereas the contribution of PdNPs is reduced in the opposite situation (M3 sample). Thus, a search for the golden combination of PdNPs and ML-Ti_3_C_2_T_*x*_ is of critical importance, as demonstrated by M2 sample. To the best of our knowledge, the aforementioned oxygen-containing groups such as C–O, C–Ti–O, and C–Ti–OH do not have any strong chemical reaction with H_2_ molecules or H atoms. Instead, they can strengthen the atomic-scale bonding between PdNPs and Ti_3_C_2_T_*x*_ MXene, thereby improving the H spill-over efficiency and H_2_ adsorption. Chung *et al.* demonstrated that the diffusion of spilt-over H atoms could be enhanced by the oxygen functional groups. They concluded that the spill-over enhancement linearly increased with the content of oxygen groups for the samples with sufficiently high oxygen concentrations.^49^

### 3.4 Hydrogen storage characteristics

**Fig. 8:**
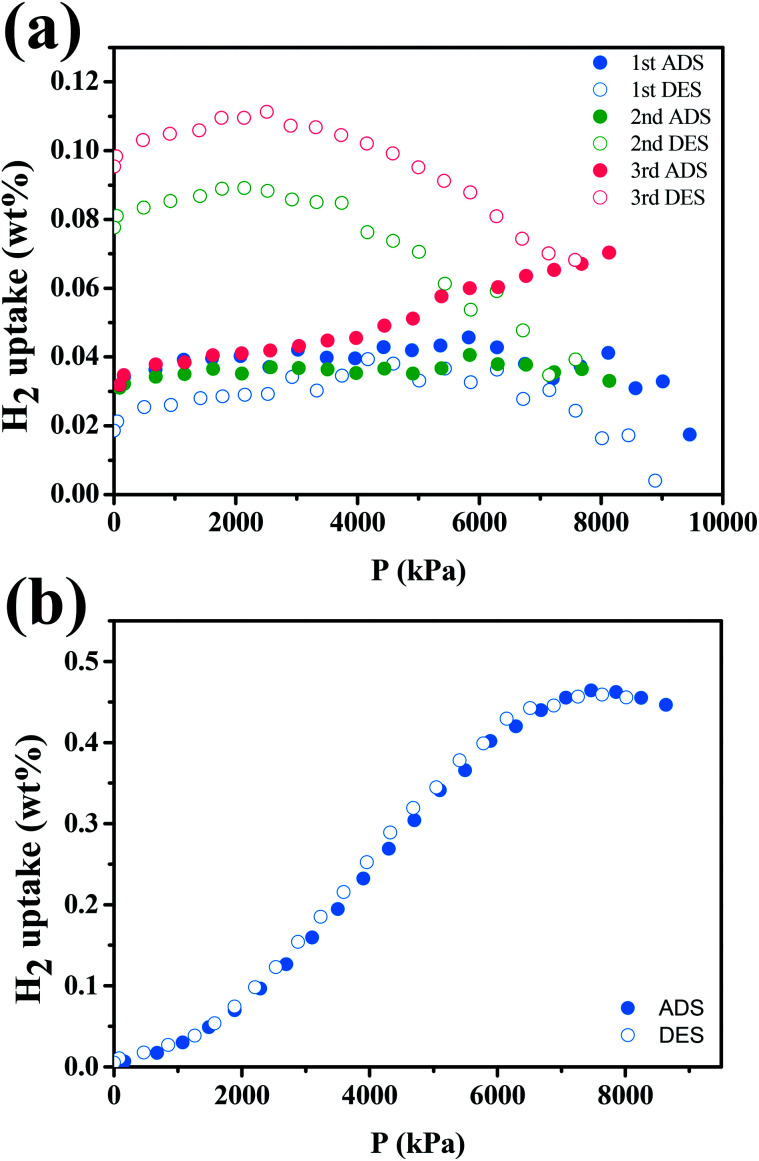
Hydrogen storage capacity of the M2 sample at (a) room temperature and (b) 77 K.

We performed hydrogen storage test on a Pd–Ti_3_C_2_T_*x*_ nanocomposite (M2 sample) by measuring the volumetric change under varying pressure at a fixed temperature. The test was taken at both room temperature and liquid nitrogen temperature (77 K). Fig. 8(a) shows the cyclic H_2_ adsorption–desorption isotherms of the sample at room temperature. The amount of stored H_2_ is small, and it tends to gradually increase as the number of cycles increases. The maximum H_2_ uptake is estimated at 0.11%. The H_2_ adsorption–desorption behavior is greatly improved at 77 K, as shown in Fig. 8(b). Clear adsorption and desorption curves are observed. Impressively, the two curves are almost superposed with negligible hysteresis, which is an ideal feature required for stable and repeated loading and disloading of H_2_. The largest H_2_ uptake is 0.46% at 7.46 MPa. Although this H_2_ uptake is far lower than those of well-developed storage media, the results suggest that the Pd–Ti_3_C_2_T_*x*_ nanocomposite can also play as a hydrogen storage. In fact, there have been rare reports on the hydrogen storage capability of Ti_3_C_2_T_*x*_ MXene. As an example, Chen *et al.* demonstrated that Ti_3_C_2_ MXene might enhance the hydrogen storage performance of MgH_2_–LiAlH_4_ composite as an ancillary material.^50^ In contrast, ML-Ti_3_C_2_T_*x*_ MXene is a main component of Pd–Ti_3_C_2_T_*x*_ for hydrogen storage. Its surface functional groups like –OH and =O are helpful for hydrogen adsorption, and surface Ti atoms may react easily with H atoms to form TiH_2_.^48^ Furthermore, PdNPs can assist the H_2_ adsorption process by the aforementioned spill-over mechanism.^51^ For these reasons, the hydrogen storage capability of the nanocomposite may be further improved.

## 4. Conclusions

Pd–Ti_3_C_2_T_*x*_ nanocomposite was synthesized by a facile two-step process. ML-Ti_3_C_2_T_*x*_ MXene was first fabricated by the HF etching of MAX phase, then PdNPs were directly decorated on the surface of ML-Ti_3_C_2_T_*x*_ using a polyol method. The average size and distribution of PdNPs were disclosed to depend on the relative weight fraction of Pd used for the PdNP formation. The material combinations of ML-Ti_3_C_2_T_*x*_ and PdNPs were tuned to find the optimal Pd–Ti_3_C_2_T_*x*_ nanocomposite. The optimal Pd–Ti_3_C_2_T_*x*_ turned out to sense H_2_ gas at room temperature with sharp, large, and concentration-dependent responses and full recovery. Furthermore, it showed high selectivity to H_2_ gas, demonstrating its potential as an ideal H_2_ gas sensor. In addition, the Pd–Ti_3_C_2_T_*x*_ nanocomposite exhibited clean and hysteresis-free H_2_ adsorption–desorption curves at 77 K, indicating that the nanocomposite could also play as a hydrogen storage.

## Conflicts of interest

The authors declare that they have no conflict of interest.

## Supplementary Material

RA-011-D0RA10879K-s001

## References

[cit1] Abe J. O., Popoola A. P. I., Ajenifuja E., Popoola O. M. (2019). Int. J. Hydrogen Energy.

[cit2] Irena I. R. E. A. , Hydrogen: A Renewable Energy Perspective – Report prepared for the 2nd Hydrogen Energy Ministerial Meeting in Tokyo, Japan, 2019

[cit3] Andersson J., Grönkvist S. (2019). Int. J. Hydrogen Energy.

[cit4] Chauhan P. S., Bhattacharya S. (2019). Int. J. Hydrogen Energy.

[cit5] Kojima Y. (2019). Int. J. Hydrogen Energy.

[cit6] Ndaya C. C., Javahiraly N., Brioude A. (2019). Sensors.

[cit7] Hu J., Sun Y., Xue Y., Zhang M., Li P., Lian K., Zhuiykov S., Zhang W., Chen Y. (2018). Sens. Actuators, B.

[cit8] Sanger A., Kumar A., Chauhan S., Gautam Y. K., Chandra R. (2015). Sens. Actuators, B.

[cit9] Harley-Trochimczyk A., Chang J., Zhou Q., Dong J., Pham T., Worsley M. A., Maboudian R., Zettl A., Mickelson W. (2015). Sens. Actuators, B.

[cit10] Zhou R., Lin X., Xue D., Zong F., Zhang J., Duan X., Li Q., Wang T. (2018). Sens. Actuators, B.

[cit11] Kandyla M., Chatzimanolis-Moustakas C., Guziewicz M., Kompitsas M. (2014). Mater. Lett..

[cit12] Martínez-Orozco R. D., Antaño-López R., Rodríguez-González V. (2015). New J. Chem..

[cit13] Koo W.-T., Qiao S., Ogata A. F., Jha G., Jang J.-S., Chen V. T., Kim I.-D., Penner R. M. (2017). ACS Nano.

[cit14] Shin D. H., Lee J. S., Jun J., An J. H., Kim S. G., Cho K. H., Jang J. (2015). Sci. Rep..

[cit15] Sinha A., Dhanjai, Zhao H., Huang Y., Lu X., Chen J., Jain R. (2018). TrAC, Trends Anal. Chem..

[cit16] Limbu T. B., Chitara B., Orlando J. D., Garcia Cervantes M. Y., Kumari S., Li Q., Tang Y., Yan F. (2020). J. Mater. Chem. C.

[cit17] Moradi R., Groth K. M. (2019). Int. J. Hydrogen Energy.

[cit18] Hirscher M., Yartys V. A., Baricco M., Bellosta von Colbe J., Blanchard D., Bowman R. C., Broom D. P., Buckley C. E., Chang F., Chen P., Cho Y. W., Crivello J.-C., Cuevas F., David W. I. F., de Jongh P. E., Denys R. V., Dornheim M., Felderhoff M., Filinchuk Y., Froudakis G. E., Grant D. M., Gray E. M., Hauback B. C., He T., Humphries T. D., Jensen T. R., Kim S., Kojima Y., Latroche M., Li H.-W., Lototskyy M. V., Makepeace J. W., Møller K. T., Naheed L., Ngene P., Noréus D., Nygård M. M., Orimo S., Paskevicius M., Pasquini L., Ravnsbæk D. B., Veronica Sofianos M., Udovic T. J., Vegge T., Walker G. S., Webb C. J., Weidenthaler C., Zlotea C. (2020). J. Alloys Compd..

[cit19] Rangel E., Sansores E., Vallejo E., Hernández-Hernández A., López-Pérez P. A. (2016). Phys. Chem. Chem. Phys..

[cit20] VahidMohammadi A. , Kayali E., Orangi J. and Beidaghi M., in 2D Metal Carbides and Nitrides (MXenes), Springer International Publishing, Cham, 2019, pp. 177–195

[cit21] Li R., Zhang L., Shi L., Wang P. (2017). ACS Nano.

[cit22] Alhabeb M., Maleski K., Anasori B., Lelyukh P., Clark L., Sin S., Gogotsi Y. (2017). Chem. Mater..

[cit23] Nguyen V. L., Nguyen D. C., Hirata H., Ohtaki M., Hayakawa T., Nogami M. (2010). Adv. Nat. Sci.: Nanosci. Nanotechnol..

[cit24] Doan T. H. P., Ta Q. T. H., Sreedhar A., Hang N. T., Yang W., Noh J.-S. (2020). ACS Sens..

[cit25] Zhu Z., Liu C., Jiang F., Liu J., Ma X., Liu P., Xu J., Wang L., Huang R. (2020). J. Hazard. Mater..

[cit26] Yuan W., Yang K., Peng H., Li F., Yin F. (2018). J. Mater. Chem. A.

[cit27] Xu L., Wu X.-C., Zhu J.-J. (2008). Nanotechnology.

[cit28] Scheibe B., Kupka V., Peplińska B., Jarek M., Tadyszak K. (2019). Materials.

[cit29] Stolaś A., Darmadi I., Nugroho F. A. A., Moth-Poulsen K., Langhammer C. (2020). ACS Appl. Nano Mater..

[cit30] Ngene P., Westerwaal R. J., Sachdeva S., Haije W., de Smet L. C. P. M., Dam B. (2014). Angew. Chem., Int. Ed..

[cit31] Nugroho F. A. A., Darmadi I., Cusinato L., Susarrey-Arce A., Schreuders H., Bannenberg L. J., da Silva Fanta A. B., Kadkhodazadeh S., Wagner J. B., Antosiewicz T. J., Hellman A., Zhdanov V. P., Dam B., Langhammer C. (2019). Nat. Mater..

[cit32] Ta Q. T. H., Tran N. M., Noh J. S. (2020). Catalysts.

[cit33] Du Y., Zhang X., Wei L., Yu B., Ma D., Ye S. (2019). Coatings.

[cit34] Halim J., Cook K. M., Naguib M., Eklund P., Gogotsi Y., Rosen J., Barsoum M. W. (2016). Appl. Surf. Sci..

[cit35] My Tran N., Thanh Hoai Ta Q., Noh J.-S. (2021). Appl. Surf. Sci..

[cit36] Zemlyanov D., Aszalos-Kiss B., Kleimenov E., Teschner D., Zafeiratos S., Hävecker M., Knop-Gericke A., Schlögl R., Gabasch H., Unterberger W., Hayek K., Klötzer B. (2006). Surf. Sci..

[cit37] Sreedhar A., Reddy I. N., Hoai Ta Q. T., Namgung G., Cho E., Noh J.-S. (2019). Ceram. Int..

[cit38] Cai Z., Park S. (2020). Sens. Actuators, B.

[cit39] Gottam S. R., Tsai C.-T., Wang L.-W., Wang C.-T., Lin C.-C., Chu S.-Y. (2020). Appl. Surf. Sci..

[cit40] Moschogiannaki M., Zouridi L., Sukunta J., Phanichphant S., Gagaoudakis E., Liewhiran C., Kiriakidis G., Binas V. (2020). Sens. Actuators, B.

[cit41] Zhao Z.-J., Ko J., Ahn J., Bok M., Gao M., Hwang S. H., Kang H.-J., Jeon S., Park I., Jeong J.-H. (2020). ACS Sens..

[cit42] Lee E., VahidMohammadi A., Yoon Y. S., Beidaghi M., Kim D.-J. (2019). ACS Sens..

[cit43] Weber M., Kim J.-Y., Lee J.-H., Kim J.-H., Iatsunskyi I., Coy E., Miele P., Bechelany M., Kim S. S. (2019). J. Mater. Chem. A.

[cit44] Yan K., Toku Y., Morita Y., Ju Y. (2018). Nanotechnology.

[cit45] Kolmakov A., Klenov D. O., Lilach Y., Stemmer S., Moskovitst M. (2005). Nano Lett..

[cit46] Peng Y., Ye J., Zheng L., Zou K. (2016). RSC Adv..

[cit47] Lupan O., Postica V., Labat F., Ciofini I., Pauporté T. (2018). Sens. Actuators, B.

[cit48] Yildirim T., Ciraci S. (2005). Phys. Rev. Lett..

[cit49] Chung T.-Y., Tsao C.-S., Tseng H.-P., Chen C.-H., Yu M.-S. (2015). J. Colloid Interface Sci..

[cit50] Chen G., Zhang Y., Cheng H., Zhu Y., Li L., Lin H. (2019). Chem. Phys..

[cit51] Kumar R., Oh J.-H., Kim H.-J., Jung J.-H., Jung C.-H., Hong W. G., Kim H.-J., Park J.-Y., Oh I.-K. (2015). ACS Nano.

