# Supplementary material for: Palladium nanoparticle-decorated multi-layer Ti3C2Tx dual-functioning as a highly sensitive hydrogen gas sensor and hydrogen storage
Source: RSC Adv. 2021 Feb 15;11(13):7492–501. doi: 10.1039/d0ra10879k (PMC8694963; doi:10.1039/d0ra10879k)
Supplement: RA-011-D0RA10879K-s001 [file RA-011-D0RA10879K-s001.pdf]

Electronic Supplementary Material (ESI) for RSC Advances.
This journal is © The Royal Society of Chemistry 2021

Supporting Information for

Palladium Nanoparticles-Decorated Multi-layer $Ti_3C_2T_x$ Dual-Functioning as Highly Sensitive Hydrogen Gas Sensor and Hydrogen Storage

Thanh Hoang Phuong Doan,[a] Won G. Hong[b] and Jin-Seo Noh*[a]

[a]*Department of Physics, Gachon University, 1342 Seongnamdaero, Sujeong-gu, Seongnam-si, Gyeonggi-do 13120, Korea*

[b]*Research Center for Materials Analysis, Korea Basic Science Institute (KBSI), Daejeon 34133, Korea*

*Author to whom correspondence should be addressed: Jin-Seo Noh: e-mail: jinseonoh@gachon.ac.kr, phone: +82 317505611

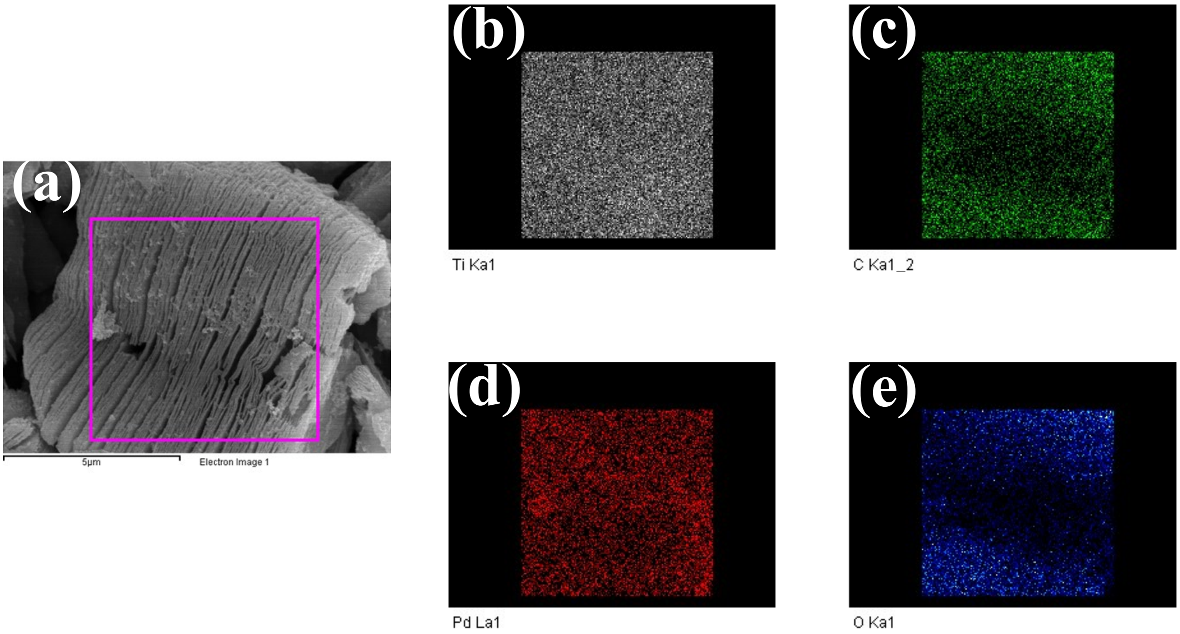

Figure S1. (a) SEM image of a Pd-$Ti_3C_2T_x$ nanocomposite (M2). SEM-EDX elemental maps of (b) Ti, (c) C, (d) Pd, and (e) O.

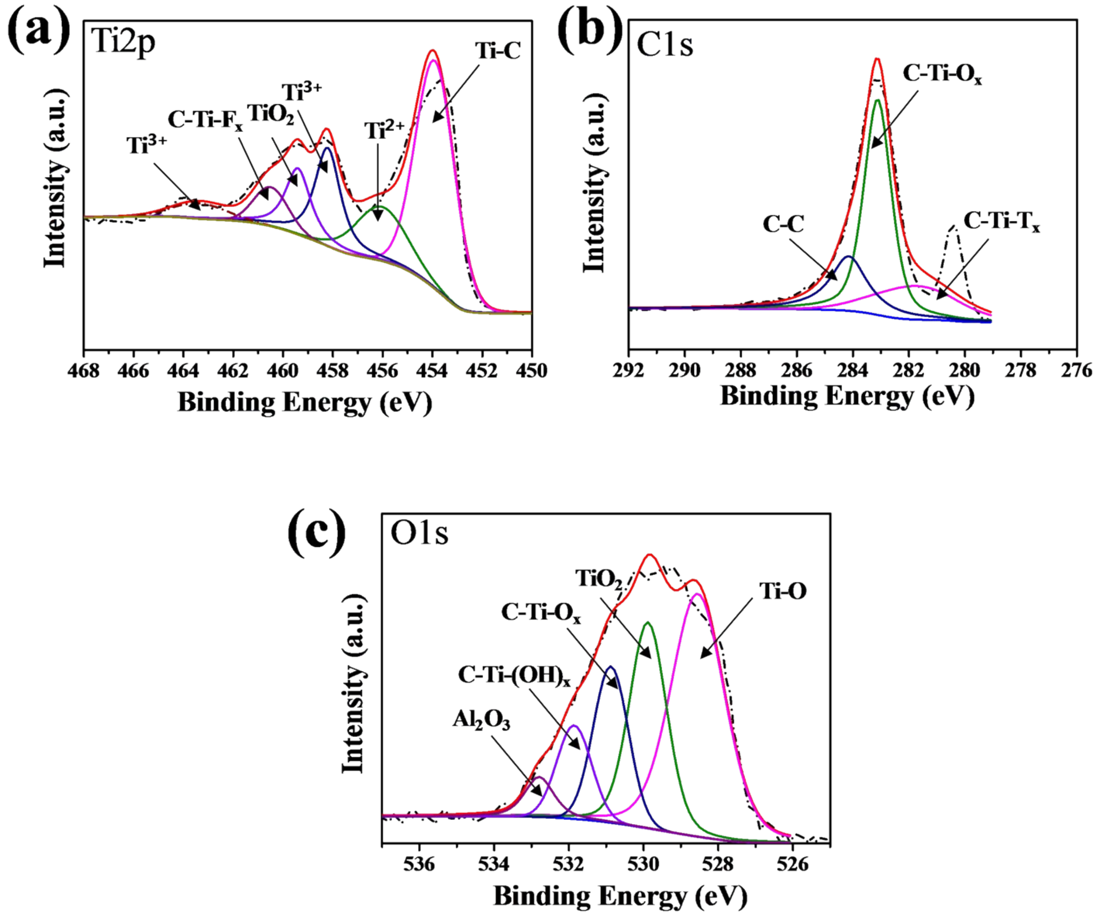

Figure S2. XPS spectra of ML-$Ti_3C_2T_x$ focused on (a) Ti, (b) C, and (c) O.

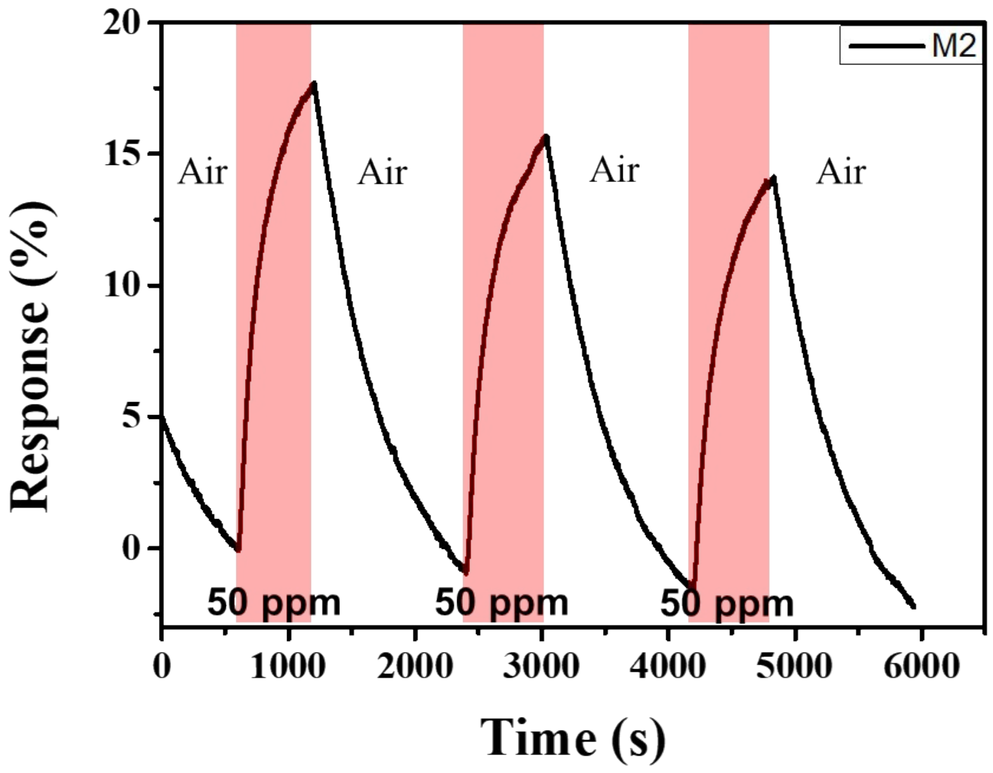

Figure S3. Cyclic responses of a Pd-$Ti_3C_2T_x$ nanocomposite (M2) to a 50 ppm of H_2 gas after keeping it for 90 days at ambient condition.
